# Improved efficacy of radiation in combination with TGFβ inhibition in a colorectal cancer mouse model

**DOI:** 10.1186/2051-1426-1-S1-P120

**Published:** 2013-11-07

**Authors:** Kristina H Young, Benjamin Cottam, Talicia Savage, Jason Baird, David Friedman, Emmanuel Akporiaye, Michael J Gough, Marka Crittenden

**Affiliations:** 1 Radiation Medicine, Oregon Health & Sciences University, Portland, OR, USA; 2 Earle A. Chiles Research Institute, Robert W. Franz Cancer Center, Providence Portland Medical Center, Portland, OR, USA

## 

Colorectal cancer patients with high levels of tumor-infiltrating T cells have better survival than patients with low levels. It is not clear whether the T cells are directly responsible for improved prognosis or are a sign of a tumor that is more responsive to conventional cancer therapies. If tumor infiltrating T cell numbers are associated with improved outcome, then we hypothesize that increasing T cell infiltrates using immunotherapy will improve the efficacy of chemoradiation. To test this hypothesis, we established CT26 colorectal carcinomas subcutaneously in immunocompetent BALB/c mice. Tumors were treated with 20Gy of radiation in a single fraction delivered using a clinical linear accelerator. To increase T cell infiltration into the tumor, an oral anti-TGFβ type I receptor small molecule inhibitor was given for one week prior to radiation. Outcomes included tumor kinetics, survival, and immune infiltrate measured by flow cytometry. TGFβ inhibition increased total T cells, activated CD8 T cells, and reduced inhibitory T regulatory cell tumor infiltrate in the tumor prior to radiation therapy. Radiation in mice pretreated with TGFβ inhibitor exhibited improved survival compared to either modality alone. In vitro clonogenic assay demonstrated equivalent radiosensitivity in control and TGFβ-inhibited cells at doses >6Gy. Small molecule penetrance measured using quantitative fluorimetry for FITC-dextran was equivalent in both treated and untreated groups. In vivo depletion of CD8 cells abrogated the efficacy of both radiation and TGFβ inhibition plus radiation. Therapy aimed at optimizing the immune environment holds promise for those colorectal cancer patients with poor immune infiltrates. Our preliminary data suggests TGFβ inhibition is a therapeutic strategy to alter tumor immune infiltrates and improve the efficacy of conventional therapies. Further studies are needed to determine the mechanism by which increased immune infiltrates improves outcome.

